# Engineering the internal surfaces of three-dimensional nanoporous catalysts by surfactant-modified dealloying

**DOI:** 10.1038/s41467-017-01085-3

**Published:** 2017-10-20

**Authors:** Zhili Wang, Pan Liu, Jiuhui Han, Chun Cheng, Shoucong Ning, Akihiko Hirata, Takeshi Fujita, Mingwei Chen

**Affiliations:** 1 WPI Advanced Institute for Materials Research, Tohoku University, Sendai, 980-8577 Japan; 2 School of Materials Science and Engineering, Shanghai Jiao Tong University, Shanghai, 200030 PR China; 3 Department of Mechanical and Aerospace Engineering, Hong Kong University of Science and Technology, Clear Water Bay, Kowloon, 999077 Hong Kong SAR; 4 CREST, Japan Science and Technology Agency, Saitama, 332-0012 Japan; 5 Department of Materials Science and Engineering, Johns Hopkins University, Baltimore, MD 21218 USA

## Abstract

Tuning surface structures by bottom-up synthesis has been demonstrated as an effective strategy to improve the catalytic performances of nanoparticle catalysts. Nevertheless, the surface modification of three-dimensional nanoporous metals, fabricated by a top-down dealloying approach, has not been achieved despite great efforts devoted to improving the catalytic performance of three-dimensional nanoporous catalysts. Here we report a surfactant-modified dealloying method to tailor the surface structure of nanoporous gold for amplified electrocatalysis toward methanol oxidation and oxygen reduction reactions. With the assistance of surfactants, {111} or {100} faceted internal surfaces of nanoporous gold can be realized in a controllable manner by optimizing dealloying conditions. The surface modified nanoporous gold exhibits significantly enhanced electrocatalytic activities in comparison with conventional nanoporous gold. This study paves the way to develop high-performance three-dimensional nanoporous catalysts with a tunable surface structure by top-down dealloying for efficient chemical and electrochemical reactions.

## Abstract (web summary)

Tuning the ratio of facets in materials can affect catalytic activity but methods to achieve this can be difficult to control. Here, using surfactants during dealloying, the authors prepare nanoporous gold with controlled facet ratios and show how it can significantly improve electrocatalytic activity.

## Introduction

The catalytic properties of heterogeneous catalysts are closely related to their terminal surfaces^1–4^. The engineering of surface structures has been demonstrated as an effective way to optimize the catalytic properties of nanostructured catalysts^1–3^. For instance, Pt_3_Ni octahedral nanoparticles bound by {111} facets show several fold higher catalytic activities toward oxygen reduction reaction (ORR) than Pt_3_Ni nanocubes with {100} facets^3^. In the past decades, the control of surface structure has been successfully realized in low-dimensional nanostructured catalysts, including nanoparticles^1, 5–9^, nanowires and nanorods^10, 11^, and thin films^12^, by bottom-up synthesis modified by organic surfactants (or capping agents, and ligands). To the best of our knowledge, tuning the surface structure of three-dimensional (3D) nanoporous catalysts, fabricated by dealloying, has not been achieved although dealloyed nanoporous catalysts have shown great promise for applications in chemical and electrochemical reactions as well as electrochemical energy convention and storage because of their unique bicontinuous structure and abundant internal surface^13–22^. Unlike low-dimensional nanostructured materials, which are usually synthesized by bottom-up chemical approaches, 3D nanoporous metals, such as nanoporous gold (NPG), are fabricated by a top-down dealloying method. In this process, less noble elements are selectively dissolved into an electrolyte from a bulk precursor alloy, while noble metal components form the skeleton of a free-standing nanoporous structure by self-assembly at metal/electrolyte interfaces^23^. The dealloyed nanoporous materials possess a 3D complex morphology with co-existent concave and convex curvatures and high curvature gradients^13, 24^. Different from nanoparticle catalysts, the selection of terminal surfaces of nanopore channels is not only determined by the surface/interface energy of crystalline ligaments but also involves surface stresses, from the confinement of precursor substrates and complicated kinetic factors during top-down dealloying. Although it is important in basic research and technical applications, tailoring the surface structure of nanoporous catalysts has not been fulfilled at the crystallographic level and it remains unknown whether the surface structure of 3D nanoporous catalysts can be controlled by the top-down dealloying to optimize their catalytic performances.

Herein, we report a surfactant-modified dealloying approach to tune the surface structure of NPG for optimal catalytic performances as a 3D nanoporous catalyst. Two types of NPG samples, pyrogallol-modified NPG (denoted as Py-NPG) enriched with {111} facets and sodium citrate-modified NPG (denoted as Na_3_CA-NPG) with an abundance of {100} facets and step/kink sites have been successfully realized. These surface-engineered NPG catalysts with preferred surface structures show a significant improvement in catalytic activities toward methanol oxidation reaction (MOR) and ORR in comparison with conventional NPG (C-NPG).

## Results

### Synthesis and characterization of surface-engineered nanoporous gold samples

**Fig. 1:**
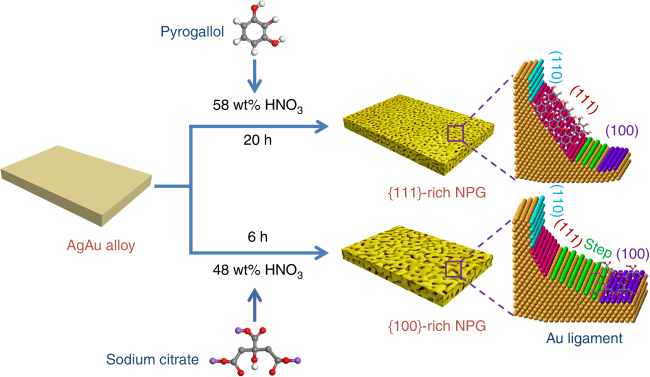
Surface engineering of 3D nanoporous catalysts. Scheme representation of the fabrication of Py-NPG and Na_3_CA-NPG

Py-NPG and Na_3_CA-NPG samples were fabricated by dealloying AgAu alloys in HNO_3_ solutions with different surfactants, as illustrated in Fig. 1. For comparison, C-NPG was also prepared without any surfactant in the dealloying electrolyte. Low-magnification scanning electron microscope (SEM, Supplementary Fig. 1) images show that the as prepared NPG samples are crack-free due to compensation of the volume changes during the removal of Ag by macroscopic shrinkage of the thin films^25^. High-magnification SEM images reveal the formation of a 3D nanoporous structure in the samples (Supplementary Fig. 2). The average diameters of nanopores/ligaments are measured to be 28.1 and 30.2 nm for Py-NPG and Na_3_CA-NPG, respectively. In contrast, without the surfactants, the C-NPG sample has a relatively larger pore size of 56.9 nm. The electric conductivities of the NPG films, measured by a standard four-probe method at room temperature, are about 1.04 × 10^7^ S m^−1^ for Py-NPG, 1.05 × 10^7^ S m^−1^ for Na_3_CA-NPG and 1.31 × 10^7^ S m^−1^ for C-NPG, which are very close to one another. The residual Ag contents in Py-NPG, Na_3_CA-NPG and C-NPG are measured by SEM energy-dispersive X-ray spectroscopy (SEM-EDS, Supplementary Fig. 3) to be 2.4, 3.7 and 1.6 at.%, respectively, well consistent with the results from inductively coupled plasma mass spectrometry (ICP-MS, Supplementary Table 1). Scanning transmission electron microscopy (STEM) coupled with EDS was used to characterize the elemental distributions of Ag and Au in these NPG samples. From the STEM-EDS elemental mappings of Au-L_α_, Ag-L_α_, and mixed-colour images of Py-NPG, Na_3_CA-NPG and C-NPG, it can be seen that the Ag and Au are uniformly distributed in the ligaments and detectable surface segregation cannot be found in all samples (Supplementary Fig. 4). The surface chemistry of the NPG samples was investigated using X-ray photoelectron spectroscopy (XPS, Supplementary Fig. 5). The Au 4 f and Ag 3d XPS spectra of the NPG samples show that the surface Au and Ag in C-NPG and Na_3_CA-NPG are mainly in the metallic state, whereas a small amount of Au and Ag on the Py-NPG surface are in the oxidized state^26^. Meanwhile, the binding energies of Au 4 f and Ag 3d in C-NPG and Na_3_CA-NPG are similar, respectively, but slightly lower than that of Py-NPG, suggesting that there is a strong interaction between gold and pyrogallol^27^. The surface molar ratios of Ag to Au are 2.8/97.2, 4.3/95.7 and 2.3/97.7 for Py-NPG, Na_3_CA-NPG and C-NPG, respectively. These values are close to their average compositions obtained from ICP-MS, further suggesting that the residual Ag is homogeneously distributed in the ligaments rather than being segregated and accumulated on the ligament surfaces.

The atomic-scale surface structures of the NPG samples were characterized using high-resolution TEM (HRTEM, Supplementary Fig. 6). From the [110] direction, {111} facets, {001} facets and atomic steps can be easily identified from the internal surfaces of three NPG samples. Clearly, Py-NPG has a much higher ratio of {111} facets than that of C-NPG and Na_3_CA-NPG. In the case of Na_3_CA-NPG, more {100} facets are observed in comparison with C-NPG and Py-NPG. These results provide straightforward evidence that Py-NPG is enriched with {111} facets whereas the Na_3_CA-NPG is abundant in {100} facets. Although HRTEM can offer atomic images on preferred surface facets in the NPG samples, it can only provide qualitative information but not quantitative fractions of each of the facets because of a very small imaging area from limited locations.

**Fig. 2:**
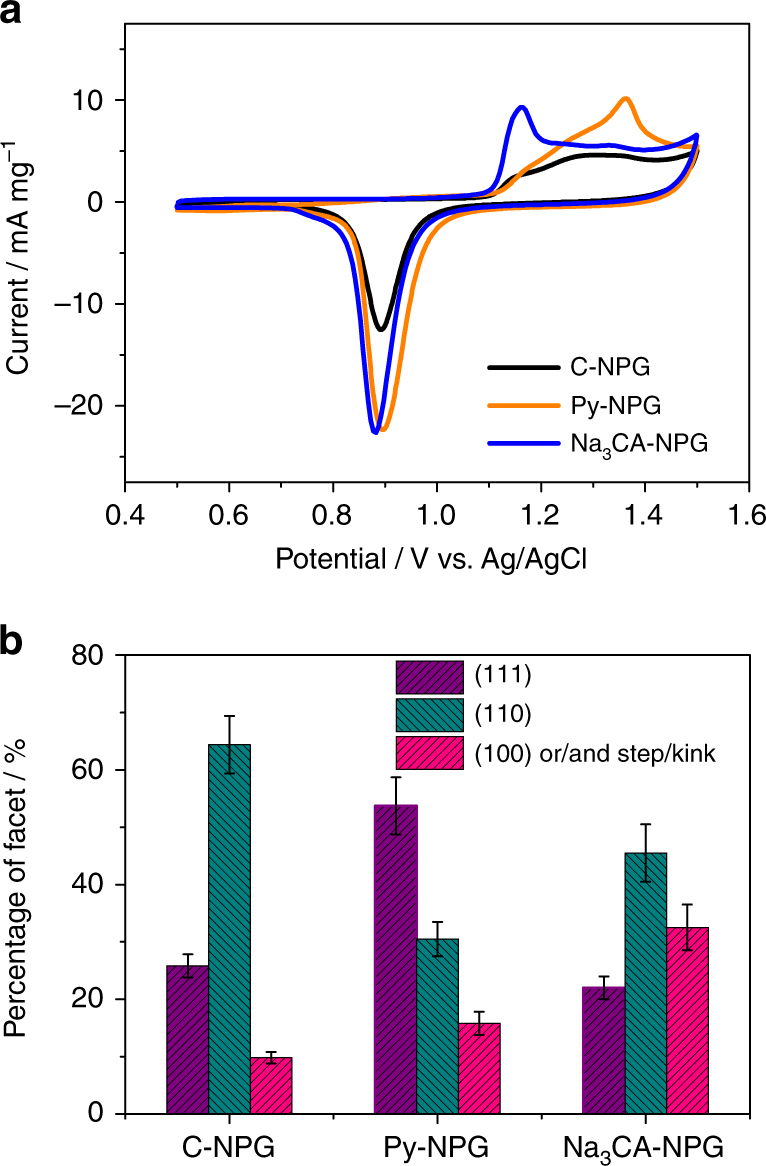
Cyclic voltammetry characterization of different NPG samples. **a** CV curves of the NPG samples recorded at room temperature in 0.1 M H_2_SO_4_ solution with a scan rate of 50 mV s^−1^. **b** The percentages of different facets in C-NPG, Py-NPG and Na_3_CA-NPG. Error bars represent standard deviations from three measurements

To quantitatively characterize the surface structure of the NPG samples, we employed cyclic voltammetry (CV) measurements and underpotential deposition methods to measure the fractions of preferred facets in each NPG sample. It is known that the electrochemical oxidation peaks in CV curves can represent specific surface facets of Au electrodes and thus have been used as fingerprints to index terminal surfaces of Au catalysts. This is because the oxide peaks are sensitive to the surface structures due to the difference of the coordination number and chemical activities of different Au facets^28, 29^. Figure 2a shows the typical CV curves (the 2nd cycle) of the C-NPG, Py-NPG and Na_3_CA-NPG in 0.1 M H_2_SO_4_ solution. The anodic peaks in the region between 1.10 and 1.50 V originate from the surface oxidation of the NPG electrodes while cathodic peaks at about 0.89 V are from the reduction of the oxidized Au surfaces^30^. The reduction peaks are insensitive to surface structures and have a near constant potential due to the occurrence of surface reconstruction and/or the formation of amorphous oxides during oxidation processes^31^. In contrast, the anodic peaks exhibit rich detail. As shown in Fig. 2a, the voltammetric profiles of three NPG samples show significant differences in the anodic behaviour. For C-NPG, a broad oxidation peak appears in a wide potential range from 1.10 to 1.44 V and a well-defined characteristic CV peak cannot be seen. In contrast, Py-NPG shows a pronounced peak at about 1.36 V from the surface oxidation of Au {111} planes^28, 29^. The CV curve of Py-NPG is also similar to those of the Au nanoparticles enclosed with {111} facets^29, 32^. Therefore, Py-NPG is predominantly enriched with {111} facets. In the case of Na_3_CA-NPG, a noticeable anodic peak is shown at about 1.14 V from the oxidation of Au {100} facets and/or dominant step/kink sites^28, 30, 33^, while the peak from the oxidation of {111} facets in Py-NPG becomes insignificant. We de-convoluted the anodic peaks to estimate the proportions of different facets on the surfaces of the NPG samples (Supplementary Fig. 7). As shown in Fig. 2b, the fractions of {111} facets, {110} facets, and {100} and/or step/kink sites in C-NPG are calculated to be 25.8, 64.4, and 9.8%, respectively. For Py-NPG, the {111} facets become the dominant surface. The fraction of {111} facets increases to 53.7% whereas the {110} facets decrease to 30.5%. The Na_3_CA-NPG exhibits a higher fraction (32.5%) of {100} facets and/or step/kink sites than that of Py-NPG (15.8%) and C-NPG. In addition, the potential and shape of the anodic peaks are almost unchanged with the scan rates (Supplementary Fig. 8), verifying that only the outermost surface atoms are oxidized and reduced during the CV measurements. Moreover, such distinct electrochemical surface features from the oxidation of specific facets also indicate that the internal surfaces of the NPG samples are clean and the surfactants have been removed by water rinsing^34^, which is intrinsically different from the surfactant-contaminated surfaces (Supplementary Fig. 9).

**Fig. 3:**
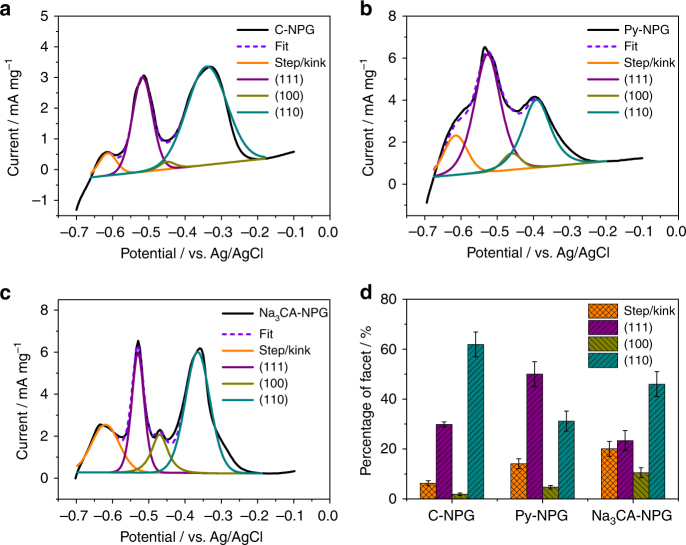
Underpotential deposition of lead on different NPG samples. Desorption voltammetric profiles of **a** C-NPG, **b** Py-NPG, and **c** Na_3_CA-NPG in 0.1 M NaOH + 10^−3^ M Pb(NO_3_)_2_. Scan rate: 50 mV s^−1^. **d** The percentages of different facets in C-NPG, Py-NPG, and Na_3_CA-NPG. Error bars represent standard deviations from three measurements

In order to further characterize the surface structure of the NPG samples, underpotential deposition of lead (Pb-upd) in alkaline media was carried out as the Pb-upd peak potentials of stripping waves are more sensitive to the types of terminal surfaces of Au electrodes in comparison with anodic oxidation^29, 35^. As displayed in Fig. 3a, the voltammetric profile of C-NPG exhibits three distinct peaks, a small peak at −0.63 V and two intense peaks at −0.52 V and −0.35 V. The small peak corresponds to the contribution of the surface step and kink sites, the intense peak at −0.52 V represents the {111} facets, and the peak at −0.35 V arises from Au {110} facets^35^. For Py-NPG, the intensity of the peak corresponding to {111} facets is much higher than that of {110} peak (Fig. 3b), further confirming that Py-NPG is predominantly enriched with {111} facets. In the case of Na_3_CA-NPG (Fig. 3c), a new stripping peak appears at −0.47 V, which is known as the characteristic of Au {100} facets^29, 35^. In addition, the intensity of the peak related to step/kink sites is higher than those measured from Py-NPG and C-NPG. Therefore, Na_3_CA indeed promotes the formation of {100} facets and step/kink sites which have the highest fraction among the three NPG samples. Because characteristic peak intensity is proportional to the surface area percentage of corresponding facets, we estimated the proportions of different facets on the internal surfaces of the NPG samples by de-convoluting the stripping waves. It should be noted that the full width at half maximum (FWHM) values of de-convolution peaks for each facet are different because the potential ranges for the dissolution of Pb from different Au facets are different^36^. As shown in Fig. 3d, the fraction of {111} facets in Py-NPG is calculated to be about 50%, whereas it is only about 23.4 and 29.9% in Na_3_CA -NPG and C-NPG, respectively. The fractions of {100} facets and step/kink sites in Na_3_CA-NPG are 10.5 and 20.1%, which are much higher than that of the Py-NPG (4.7 and 14.1%) and C-NPG (1.9 and 6.3%). Meanwhile, the fractions of {110} facets in Py-NPG and Na_3_CA-NPG decrease from 61.9% of C-NPG to 31.2 and 46%, respectively. The Pb-upd results are fairly consistent with those from the CV measurements. Apparently, the surface structure of NPG can be tailored by using pyrogallol and Na_3_CA as the structure-directing agents during dealloying.

Although dealloying is a top-down process that selectively etches a bulk alloy precursor, the formation of nanopores is via layer-by-layer self-deposition of Au atoms from the topmost surface to the interior of the bulk precursor^23^. Appropriate surfactants are expected to moderate the surface energy and thus alter the diffusion kinetics and deposition rate of Au atoms by selective adsorption on specific crystal facets during Au ligament formation and growth^37^. Actually, our results are consistent with the fact that pyrogallol molecules and citrate ions bind more strongly to {111} and {100} facets of noble metal surfaces^37, 38^. Thus, we believe that the pyrogallol and Na_3_CA preferentially adsorb on the {111} facets and {100} facets, respectively, under optimized dealloying conditions and lead to the formation and stabilization of {111} and {100} facets. To understand the preferred facet growth mechanism, the evolution of different facets in Py-NPG and Na_3_CA-NPG with dealloying time is measured by Pb-upd (Supplementary Figs. 10 and 11). At the initial stage of dealloying, the {110} facets are the preferentially formed surface, even in the presence of pyrogallol or Na_3_CA, which is similar to C-NPG dealloyed without any surfactant. For Py-NPG, with the dealloying time increasing, the fraction of {111} facets clearly increases while the fraction of {110} facets decreases and {100} and steps/kinks only have a slight increase. Apparently, under the optimized dealloying conditions, pyrogallol preferentially stabilizes the {111} planes of Au ligaments and, in contrast, {110} facets with a high surface energy gradually change to the {111} planes possibly by surface self-deposition or reconstruction, similar to faceted gold nanoparticles^6^. For Na_3_CA-NPG, the fractions of {100} facets and steps/kinks increase while the fractions of {110} facets and {111} facets decrease with increasing dealloying time. Similar to the growth mechanism of {111} facets in Py-NPG, Na_3_CA preferentially stabilizes the {100} planes of Au ligaments and consequently the transition from {110} to {100} facets is promoted. Meanwhile, the transition from {110} to {111} facets is prevented and a small portion of {111} facets even transforms into {100} facets and steps/kinks during dynamic reconstruction of ligament surfaces. On the other hand, when pyrogallol or Na_3_CA was substituted with other surfactant, such as (poly)vinpyrrolidone (PVP) which has been a widely used as a structure-directing agent for the synthesis of {111}-faceted Au nanoparticles, the obtained NPG sample has a lower fraction of {111} facets than that of Py-NPG (Supplementary Fig. 12), highlighting the unique function of pyrogallol and Na_3_CA in controlling the surface structure of dealloyed NPG. Besides the selection of surfactants, the concentration of surfactants is also a critical factor in tailoring the surface structure of NPG. For both pyrogallol and Na_3_CA, there is an optimal concentration to maximize the fraction of {111} facets or {100} facets and step/kink sites. The optimal concentration of pyrogallol is about 0.2 mg mL^−1^, which gives the largest fraction of {111} facets of about 50%. Increasing or decreasing the pyrogallol concentration to above or below 0.2 mg mL^−1^ results in the decrease of the fraction of {111} facets (Supplementary Fig. 13). For Na_3_CA-NPG, the fractions of {100} facets and step/kink sites also change with the concentration of Na_3_CA and show a maximum at about 2.0 mg mL^−1^ (Supplementary Fig. 14). The optimal concentrations may correspond to a balance point. Above the optimal concentration, excessive surfactant molecules may start to bind on nonspecific facets while below the point the number of surfactant molecules is not sufficient to stabilize all specific facets^37^. Therefore, both situations lead to the decrease of the preferred facets. Besides the selection of the surfactant, we noticed that the formation of preferred surface facets is affected by the precursor alloy compositions and acid concentrations, which are clearly different from the bottom-up methods. The combination of pyrogallol with the Ag_65_Au_35_ precursor gives a much higher fraction of {111} facets (50%) than that with Ag_75_Au_25_ (28.6%). In contrast, the Na_3_CA works well with the Ag_75_Au_25_ precursor for the formation of a high fraction of {100} facets (10.5%) but only gains 3.1% {100} facets after the precursor is changed to Ag_65_Au_35_ (Supplementary Fig. 15). Similar to the precursor composition effect, the formation of specific facets is also influenced by the acid concentration. In our study, we used 48 and 58 wt% HNO_3_ solutions to fabricate Py-NPG and Na_3_CA-NPG and found that the activities of surfactants can be affected by the acid concentrations. For Py-NPG, 58 wt% HNO_3_ yields an obviously higher fraction of {111} facets than 48 wt% HNO_3_. In contrast, Na_3_CA-NPG has a higher fraction of {100} facets in 48 wt% HNO_3_ solution (Supplementary Fig. 16). Additionally, a slower dealloying rate benefits the formation of preferred facets by minimizing the effects of surface stress, precursor strain and dealloying kinetics in facet selection. This was confirmed by the fact that the Py-NPG samples dealloyed at higher temperature and therefore at higher dealloying rate, have a lower fraction of {111} facets. As shown in Supplementary Fig. 17, the percentage of {111} facets decreases from 50 to 39%, 36.9 and 35% with the increase of the dealloying temperature from room temperature (22 °C) to 40, 50 and 60 °C. In the top-down fabrication process, pyrogallol and Na_3_CA are not only the structure-directing agents to tune the surface structure but also act as stabilizers to prevent the coarsening of 3D nanoporous structure during dealloying. Therefore, the average pore sizes of Py-NPG decrease from 33.3, 28.1, 24.4 to 20.3 nm with the increase of the concentration of pyrogallol from 0.1 to 0.2, 0.4, and 0.8 mg mL^−1^ (Supplementary Fig. 18).

### Electrochemical performance of surface-engineered nanoporous gold

**Fig. 4:**
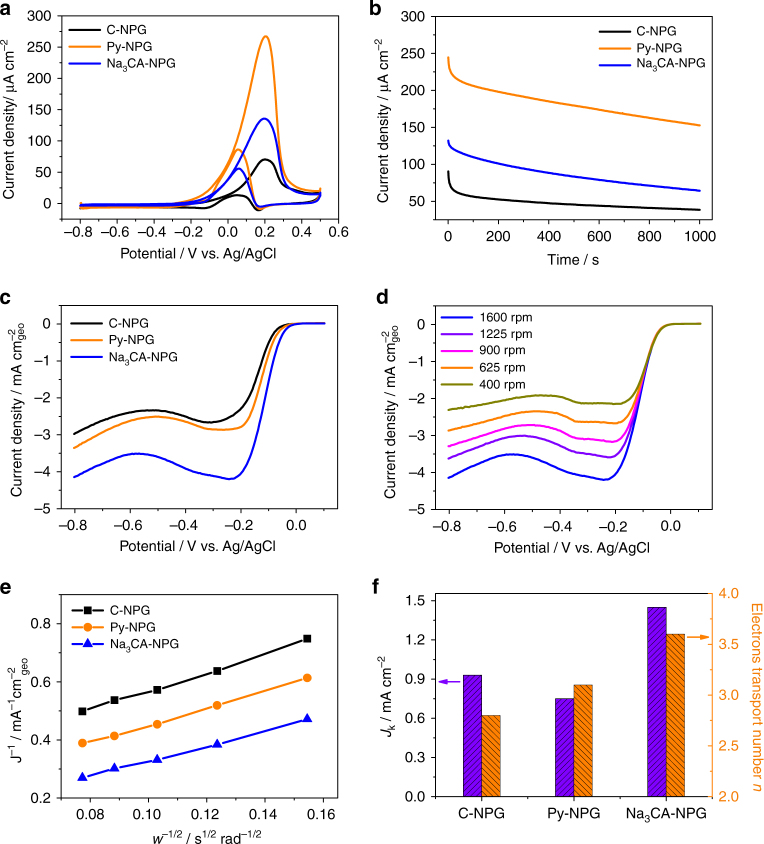
Electrochemical performance of surface-engineered NPG. **a** CV curves of MOR on Py-NPG, Na_3_CA-NPG, and C-NPG in 0.5 M KOH/1.0 M methanol solution (scan rate: 10 mV s^−1^). **b** Chromoamperometric results of MOR at 0.2 V on Py-NPG, Na_3_CA-NPG, and C-NPG in 0.5 M KOH/1.0 M methanol solution. **c** ORR polarization curves recorded in an O_2_ saturated 0.1 M KOH with a sweep rate of 10 mV s^−1^ and a rotation rate of 1600 rpm. **d** ORR polarization curves of Na_3_CA-NPG at different rotation speeds. **e** The corresponding Koutecky-Levich plots of different samples at −0.175 V. **f** Kinetic limiting current (*J*_*k*_) of different NPG samples and the corresponding electron transfer numbers (n) at −0.175 V

It has been demonstrated that the catalytic performance of NPG is closely related to its surface structure^13^. We investigated the correlation between surface structure and catalytic activity of NPG toward MOR and ORR. Figure 4a depicts the CVs of Py-NPG, Na_3_CA-NPG and C-NPG in 0.5 M KOH with 1.0 M methanol. In order to directly compare the catalytic activities of the NPG samples, the electrode current densities are normalized by the electrochemically active surface areas (ECSAs). The ECSAs of Py-NPG, Na_3_CA-NPG and C-NPG were calculated to be 8.1, 7.9 and 4.6 m^2^ g^−1^, respectively (Supplementary Table 2), by assuming that the charges associated with the reduction of oxide species are 450 µC cm^−2^ for Au surfaces^39^. The characteristic methanol oxidation peaks are identified in the forward and backward scans^40^. The peak current density of Py-NPG in the forward sweep is 267.3 µA cm^−2^, which is nearly 1.9 and 3.8 times higher than that of the Na_3_CA-NPG (135.8 µA cm^−2^) and C-NPG (70.3 µA cm^−2^), respectively. Importantly, the onset potential of Py-NPG for MOR shifts negatively to −0.29 V from −0.18 V of C-NPG, demonstrating that Py-NPG with rich {111} facets is much more active in catalyzing methanol oxidation than C-NPG. The higher methanol oxidation current density of Py-NPG is further confirmed by chronoamperometric (CA) measurements performed at 0.2 V for 1000 s (Fig. 4b). The CA curves show that the current density of Py-NPG is noticeably higher than that of Na_3_CA-NPG and C-NPG in the entire testing period. It is worth noting that Py-NPG exhibits the highest specific current density among reported Au catalysts, including trisoctahedral gold nanoparticles (178 µA cm^−2^)^40^, hollow nanoporous gold nanoparticles (117 µA cm^−2^)^39^, branched Au nanostructures (103 µA cm^−2^)^41^, nanoporous gold film (103 µA cm^−2^)^42^, and spherical Au nanoparticles (65 µA cm^−2^)^42^, for MOR under the same experimental conditions. Since the catalytic activities of NPG also depend on pore sizes^17, 18^, to clarify possible pore size effect in the high catalysis of Py-NPG, we prepared two additional C-NPG samples with smaller pore sizes of 11.2 (C-NPG_11_) and 17.1 nm (C-NPG_17_) to test the catalytic activities (Supplementary Fig. 19). Both C-NPG_11_ and C-NPG_17_ show improved catalytic properties compared with C-NPG with a pore size of 56.9 nm. However, although the pore sizes of C-NPG_11_ and C-NPG_17_ are smaller than those of the surfactant-modified NPG samples, their catalytic performances are clearly inferior to those of Py-NPG and Na_3_CA-NPG (Supplementary Fig. 20). Thus, the significant enhancement in the catalytic activities of Py-NPG and Na_3_CA-NPG is mainly due to the modified surface structure. Considering that the Py-NPG and C-NPG_17_ contain a similar amount of residual Ag but Py-NPG exhibits much higher catalytic activities than C-NPG_17_, the residual Ag is apparently not the key factor in the enhanced catalysis. Previous studies have shown that MOR in alkaline solutions is sensitive to the surface facets of Au catalysts, and Au {111} and step/kink sites give higher catalytic activities than those of {100} and {110} facets^43, 44^. The current observations from 3D nanoporous catalysts are well in line with these results from nanoparticle Au catalysts. In addition, there were no significant changes in the fractions of the preferred facets after CA measurements, suggesting high stability of the surface optimized NPG catalysts (Supplementary Fig. 21 and Supplementary Table 3).

The ORR polarization curves of the NPG samples were measured in an O_2_ saturated 0.1 M KOH solution at 1600 rpm (Fig. 4c). The Na_3_CA-NPG sample displays the highest ORR onset potential of −0.08 V, which is much more positive than Py-NPG (−0.11 V) and C-NPG (−0.13 V). The ORR current of Na_3_CA-NPG is about two times higher than that of C-NPG over the entire potential range. Figure 4d and Supplementary Fig. 22 show the polarization curves of ORR measured by a rotating disk electrode at rotation rates ranging from 400 to 1600 rpm. The Koutecky-Levich plots of the NPG samples are presented in Fig. 4e. The number of transferred electrons (*n*) for Na_3_CA-NPG is calculated to be 3.6 at −0.175 V from the slopes of the Koutecky-Levich plots (Fig. 4f), suggesting that Na_3_CA-NPG exhibits a dominant four-electron oxygen reduction process. In contrast, the numbers of transferred electrons for Py-NPG and C-NPG at the same potential is only 3.1 and 2.8, respectively. The kinetic limiting current (*J*_k_) for Na_3_CA-NPG is calculated according to the intercept of the Koutecky-Levich plots at −0.175 V and compared with the Py-NPG and C-NPG (Fig. 4f). Significantly, the *J*_k_ value of Na_3_CA-NPG (1.45 mA cm^−2^) is much higher than that of Py-NPG (0.75 mA cm^−2^) and C-NPG (0.93 mA cm^−2^), consistent with the previous reports that the most active single-crystal electrode for ORR in alkaline media is Au {100}, whereas Au {111} is the least active plane^45^. In addition, the step/kink sites were also found to be more active than the Au {111} facets for ORR^29^. Although the smaller pore sizes of NPG can enhance the ORR activities, the pore size effect is insignificant in comparison with the selective formation of ORR-active {100} facets in Na_3_CA-NPG with a similar pore size (Supplementary Fig. 23). Although the residual Ag is found to more or less affect the ORR activity of NPG, the fact that the NPG samples with higher (C-NPG_9_ and C-NPG_11_) and lower (C-NPG_17_ and Py-NPG) residual Ag concentrations than Na_3_CA-NPG all exhibit much lower ORR activities than Na_3_CA-NPG (Supplementary Figs. 19 and 23) suggests that the effect of residual Ag on ORR activities of NPG is minor in comparison with that of the preferred {100} facets. Thus, the improved ORR activity of the Na_3_CA-NPG can be ascribed to the multitude of {100} facets and step/kink sites by surfactant-modified dealloying.

## Discussion

In summary, we have successfully developed a surfactant-modified dealloying approach to tailor the surface structure of 3D NPG. By selecting the surfactants and optimizing the dealloying parameters, the internal surface structure of the porous catalyst can be engineered to realize the preferential surface facets for MOR and ORR, respectively, with enhanced electrocatalysis. This work not only opens up an avenue to improve the electrocatalytic performance of 3D nanoporous catalysts by surface engineering, but also provides a research direction in which to develop 3D nanoporous catalysts with controllable surface structure for energy and environment related chemical and electrochemical reactions.

## Methods

### Chemicals

Pyrogallol (C_6_H_3_(OH)_3_, > 99%), sodium citrate hydrate (C_6_H_5_Na_3_O_7_·2H_2_O, > 99%), polyvinpyrrolidone (PVP, > 99%), methanol (CH_3_OH, > 99.8%), sodium hydroxide (NaOH, > 97%), potassium hydroxide (KOH, > 85%), lead (II) nitrate (Pb(NO_3_)_2_, > 99.9%), and nitric acid (HNO_3_, 69%) were purchased from Wako Pure Chemical Industries, Ltd. All chemical were used as received without further purification. Deionized water with a specific resistance of 18.2 MΩ cm was obtained by reverse osmosis followed by ion exchange and filtration.

### Fabrication of Py-NPG, Na_3_CA-NPG and C-NPG

For preparation of Py-NPG, the Ag_65_Au_35_ (at.%) leaves with a thickness of approximately 100 nm were handled by a glass sheet and floated on 50 mL 58 wt% HNO_3_ solution containing pyrogallol (0.2 mg mL^−1^) for 20 h at room temperature. For preparation of Na_3_CA-NPG, the Ag_75_Au_25_ (at.%) leaves with a thickness of approximately 100 nm were handled by a glass sheet and floated on 50 mL 48 wt% HNO_3_ solution containing Na_3_CA (2.0 mg mL^−1^) for 6 h at room temperature. For preparation of C-NPG, the Ag_65_Au_35_ (at.%) leaves with a thickness of approximately 100 nm were handled by a glass sheet and floated on 50 mL 58 wt% HNO_3_ solution for 3 h at room temperature. The dealloying periods and electrolytes listed here are the optimal conditions based on a series of experiments for the most significant surface modifications. The obtained NPG samples were carefully rinsed with distilled water to remove residual acid, surfactants and impurity ions.

### Fabrication of C-NPG_9_, C-NPG_11_ and C-NPG_17_

The Ag_65_Au_35_ (at.%) leaves with a thickness of approximately 100 nm were handled by a glass sheet and floated on 50 mL 58 wt% HNO_3_ solution at the temperature of about 1 °C for 30 min (C-NPG_11_) and 2 h (C-NPG_17_), respectively. C-NPG_9_ sample was prepared by selectively etching Ag from Ag_65_Au_35_ (at.%) leaves by using 69 wt% HNO_3_ at room temperature for 6 min. The obtained NPG samples were carefully rinsed with distilled water to remove the residual acid and impurity ions.

### Microstructural and chemical characterization

The microstructure and chemical composition of the NPG samples were investigated using a field-emission scanning electron microscope (JEOL JIB-4600F, 15 keV) equipped with an X-ray energy-dispersive spectrometer. The pore sizes of the NPG samples were determined by rotational fast Fourier transform (FFT)^46^. A JEOL JEM-2100F transmission electron microscope with an acceleration voltage of 200 kV was employed to characterize the surface elemental distributions and surface structures of NPG. The surface chemistry of the NPG samples was also investigated by using X-ray photoelectron spectroscopy (XPS, AxlS-ULTRA-DLD) with an Al Ka (mono) anode at energy of 150 W. Inductively coupled plasma-mass spectrometry (ICP-MS) measurements were performed using a Finnigan ELEMENT XR double focusing magnetic sector field ICP-MS.

### Electrochemical measurements

All electrochemical measurements were conducted in a three-electrode configuration with a Pt foil serving as the counter electrode and an Ag/AgCl electrode as the reference electrode at room temperature. The surface areas of the NPG electrodes used for CV, Pb-upd and MOR measurements are 0.072 cm^−2^. The very close loading masses of the NPG samples used for CV, Pb-upd and MOR measurements were about 5.7 µg for Na_3_CA-NPG, 5.9 µg for C-NPG, and 6.0 µg for Py-NPG. CV measurements were carried out in an Ar-saturated 0.1 M H_2_SO_4_ solution. Only the second cycle curves were used to estimate the fractions of facets in all samples to avoid possible influence of residual organic adsorbents for the redox reactions in the first cycle. For MOR, the solutions were deoxygenated by bubbling with high pure Argon for more than 20 min prior to each measurement. CV curves were recorded by using 0.5 M KOH solution with 1.0 M methanol (scan rate: 10 mV s^−1^). Chromoamperometric curves were recorded by using a 0.5 M KOH solution with 1.0 M methanol at 0.2 V. ORR measurements were conducted in O_2_-saturated 0.1 M KOH solution, which was purged with O_2_ during the measurement. The surface areas of the NPG electrodes used for ORR measurements were 0.196 cm^−2^. The loading masses of the NPG samples used for ORR measurements were about 15.5 µg for Na_3_CA-NPG, 16.0 µg for C-NPG, and 16.3 µg Py-NPG. The rotating disk electrode experiments were carried out in O_2_-saturated 0.1 M KOH with a sweep rate of 10 mV s^−1^ and at varying speeds (400–1600 rpm).

### Data availability

The data that support the findings of this study are available from the authors upon request.

## Electronic supplementary material

Supplementary Information
