# Supplementary material for: Engineering the internal surfaces of three-dimensional nanoporous catalysts by surfactant-modified dealloying
Source: Nat Commun. 2017 Oct 20;8:1066. doi: 10.1038/s41467-017-01085-3 (PMC5651939; doi:10.1038/s41467-017-01085-3)
Supplement: Supplementary file 1 — Supplementary Information [file 41467_2017_1085_MOESM1_ESM.pdf]

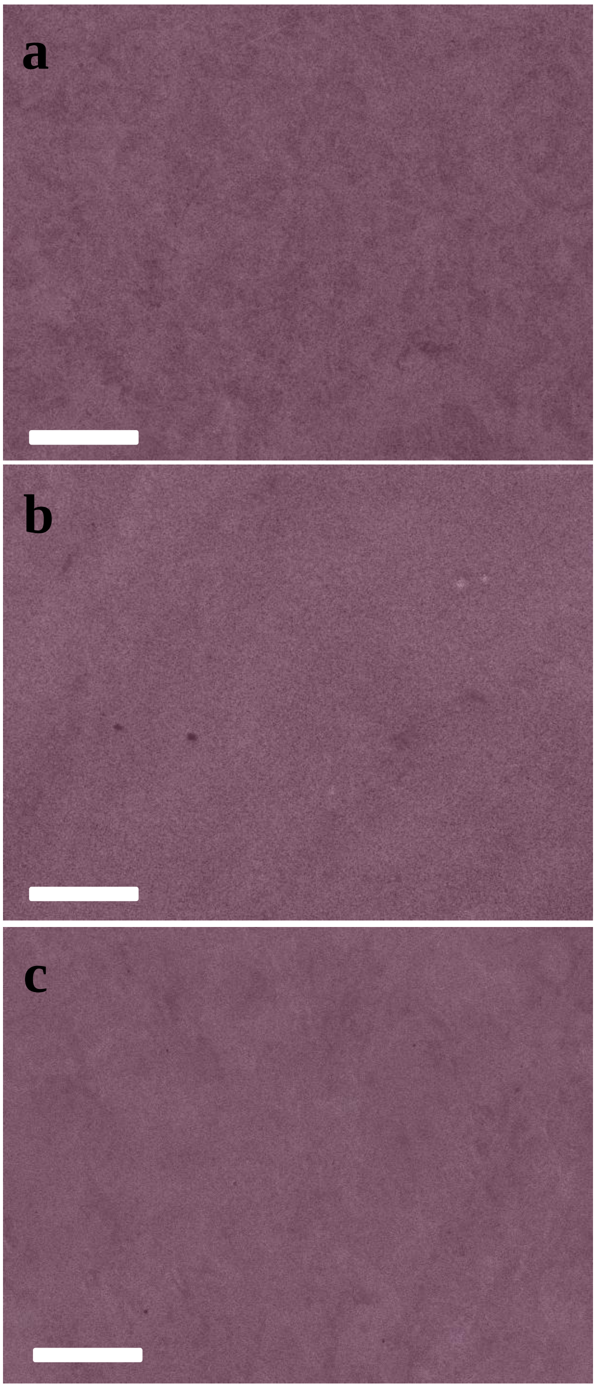

Supplementary Figure 1. Low-magnification SEM images of (a) Py-NPG, (b) Na_3CA-NPG and (c) C-NPG. Scale bars: 10 μm.

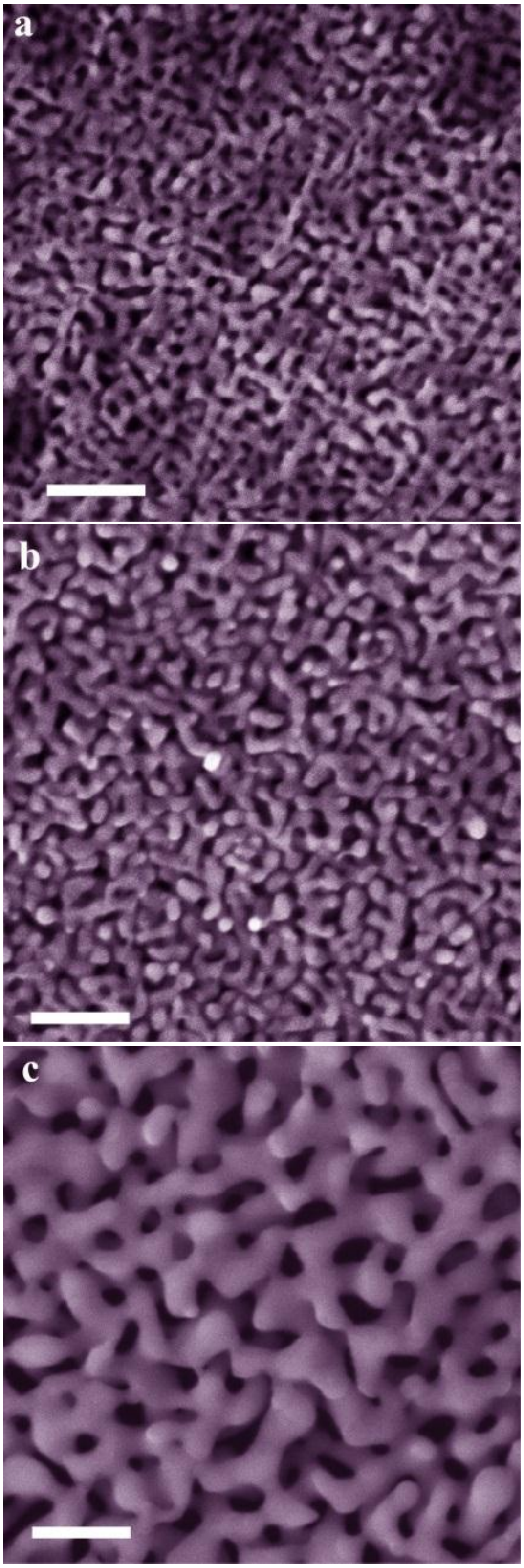

Supplementary Figure 2. High-magnification SEM images of (a) Py-NPG, (b) Na_3CA-NPG, and (c) C-NPG. Scale bars: 200 nm.

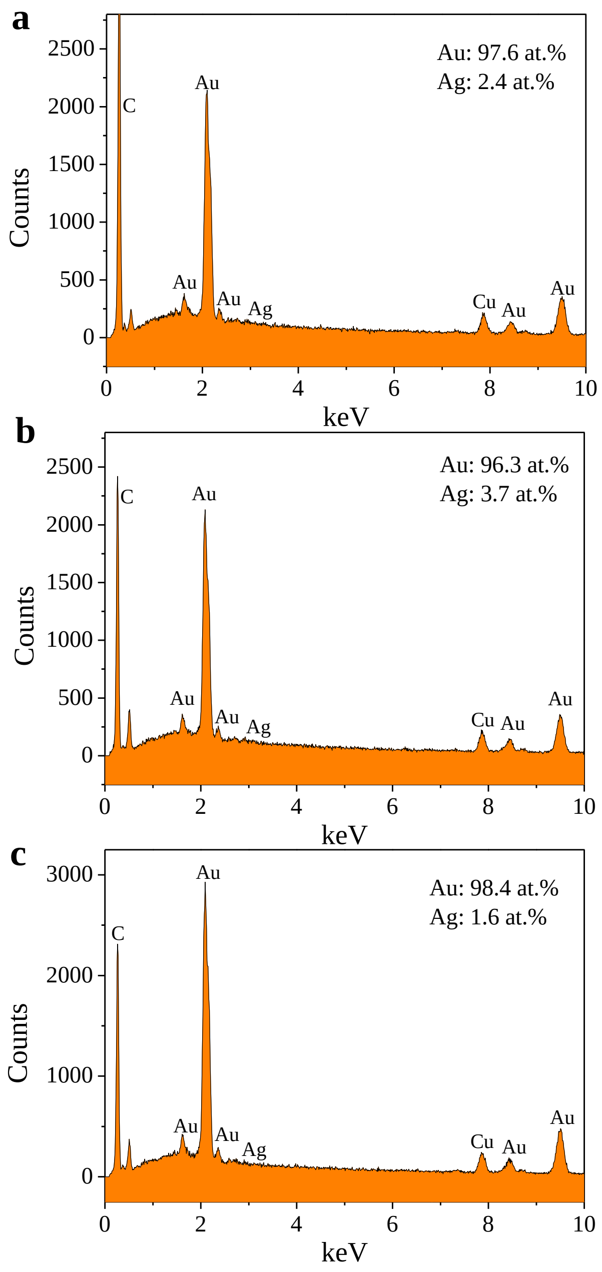

Supplementary Figure 3. Energy dispersive X-ray spectroscopy (EDS) spectra of (a) Py-NPG, (b) Na_3CA-NPG and (c) C-NPG. The Cu peaks are from the copper sample holder.

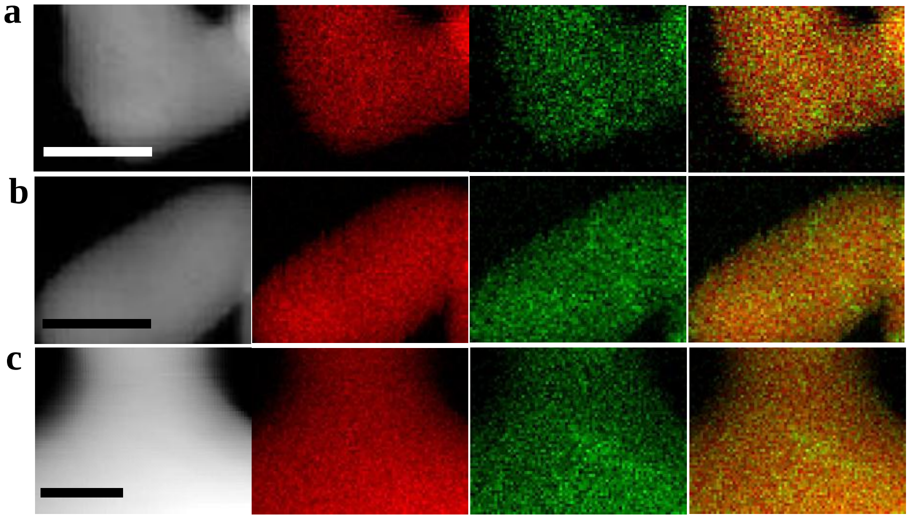

Supplementary Figure 4. STEM images and corresponding EDS elemental mapping of Au-$L_α$ (in red), Ag-$L_α$ (in green), and mixed-color images of (a) Py-NPG, (b) Na_3CA-NPG and (c) C-NPG. Scale bars: 20 nm.

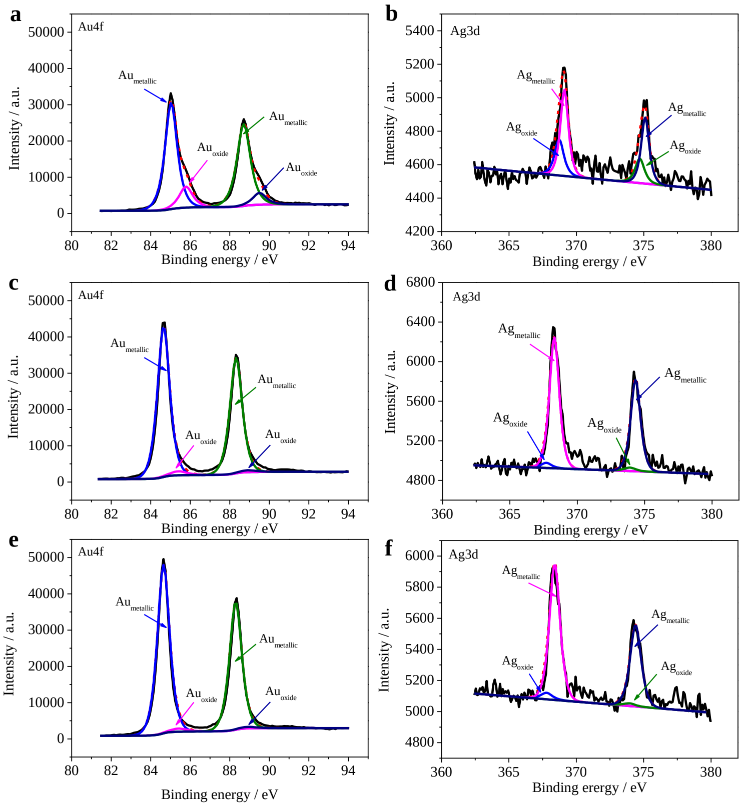

Supplementary Figure 5. Au 4f and Ag 3d XPS spectra of (a,b) Py-NPG, (c,d) Na_3CA-NPG and (e,f) C-NPG.

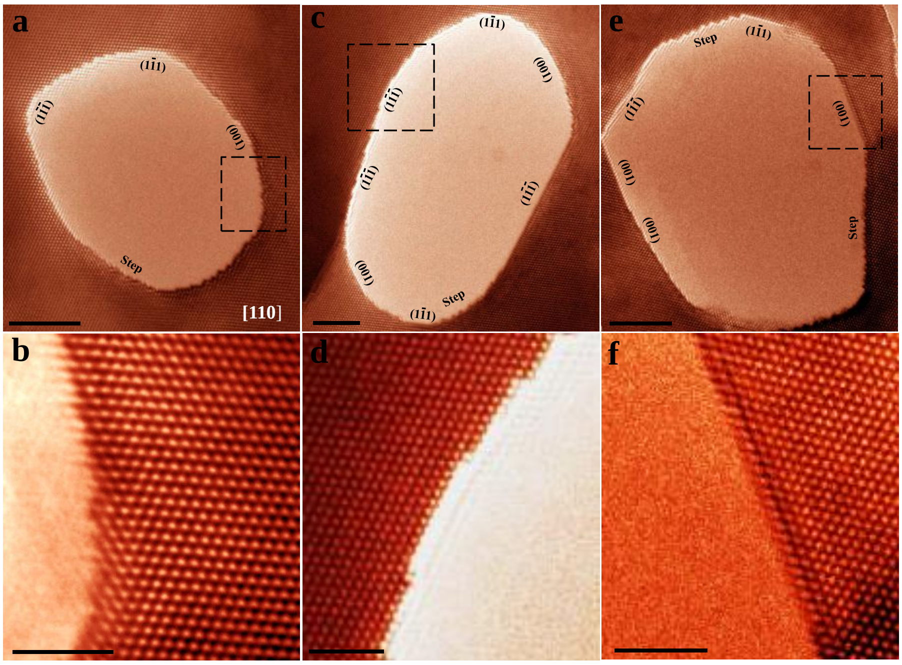

Supplementary Figure 6. HRTEM images of (a, b) C-NPG, (c, d) Py-NPG and (e, f) Na_3CA-NPG viewed along [110]. Scale bars in images of (a,c,e) and (b,d,f) are 5 nm and 2 nm, respectively.

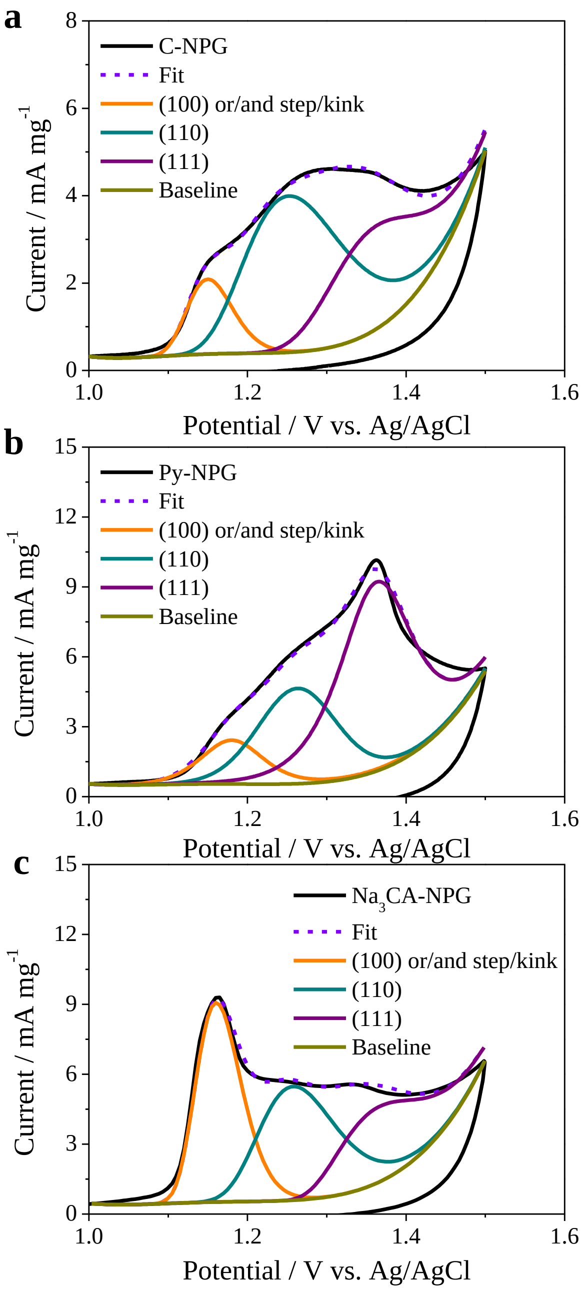

Supplementary Figure 7. Deconvoluted CV curves of (a) C-NPG, (b) Py-NPG, and (c) Na_3CA-NPG in the region 1.00-1.50 V. The baselines are set based on the charges for the oxidation of NPG electrodes equal to the charges for the reduction of the oxidized Au. The FWHM values of deconvolution peaks for each facet are different because the potential ranges for the oxidation of different Au facets are different.

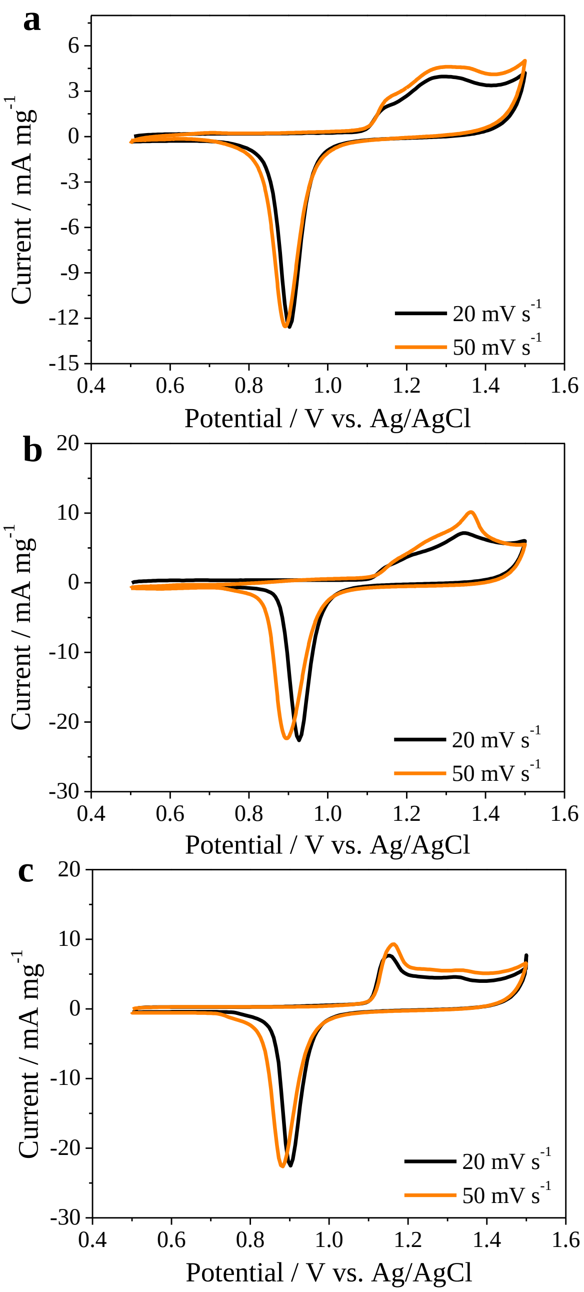

Supplementary Figure 8. CV curves of (a) C-NPG, (b) Py-NPG, and (c) Na_3CA-NPG recorded at room temperature in 0.1 M H_2SO_4 solution with different scan rates. For comparison, the currents of the NPG samples obtained with low scan rate are enlarged to the same as the currents obtained with the high scan rate.

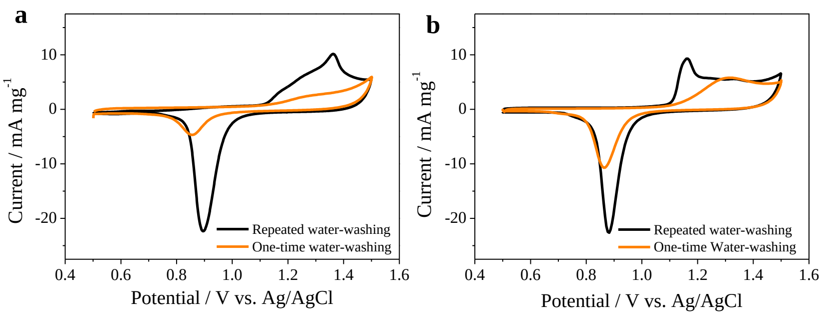

Supplementary Figure 9. CV curves of (a) Py-NPG and (b) Na_3CA-NPG after repeated or one-time water-washing in 0.1 M H_2SO_4 solution at room temperature with a scan rate of 50 mV s^{-1}.

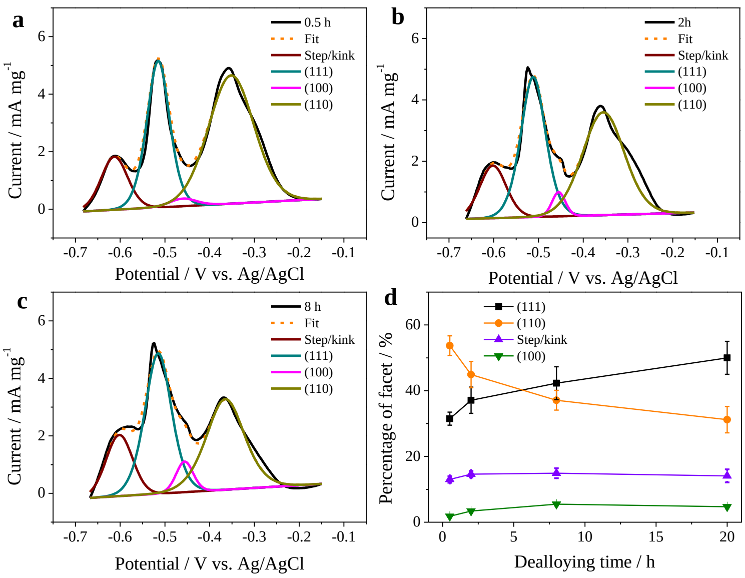

Supplementary Figure 10. (a-c) Desorption voltammetric profiles of Py-NPG in 0.1 M NaOH + 10^{-3} M $Pb(NO_3)_2$ at different dealloying time. Scan rate: 50 mV s^{-1}. (d) The evolution of facet fractions with dealloying time. Error bars represent standard deviations from three measurements.

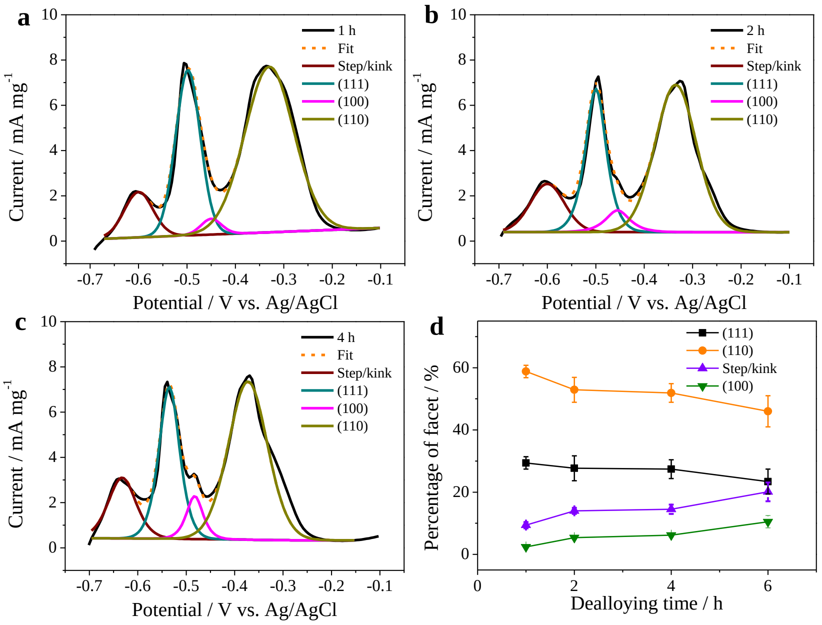

Supplementary Figure 11. (a-c) Desorption voltammetric profiles of Na_3CA-NPG in 0.1 M NaOH + 10^{-3} M $Pb(NO_3)_2$ at different dealloying time. Scan rate: 50 mV s^{-1}. (d) The evolution of facet fractions with dealloying time. Error bars represent standard deviations from three measurements.

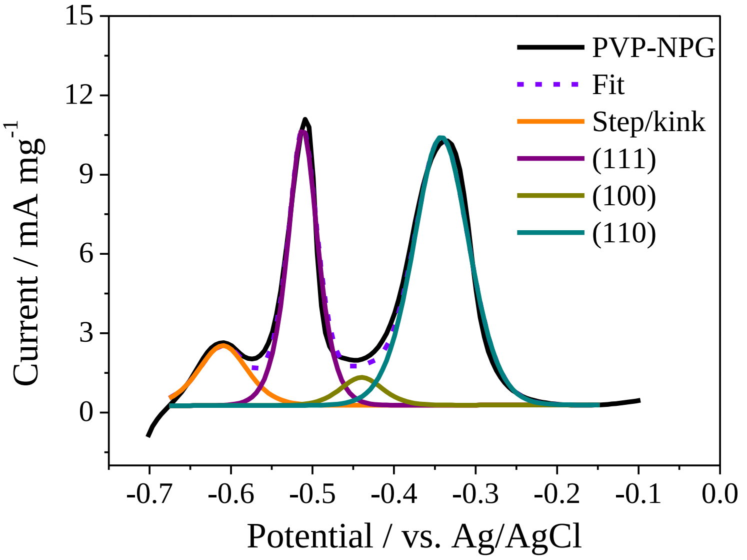

Supplementary Figure 12. Desorption voltammetric profile of PVP-NPG prepared with 1.5 mg mL^{-1} PVP in 0.1 M NaOH + 10^{-3} M $Pb(NO_3)_2$. Scan rate: 50 mV s^{-1}.

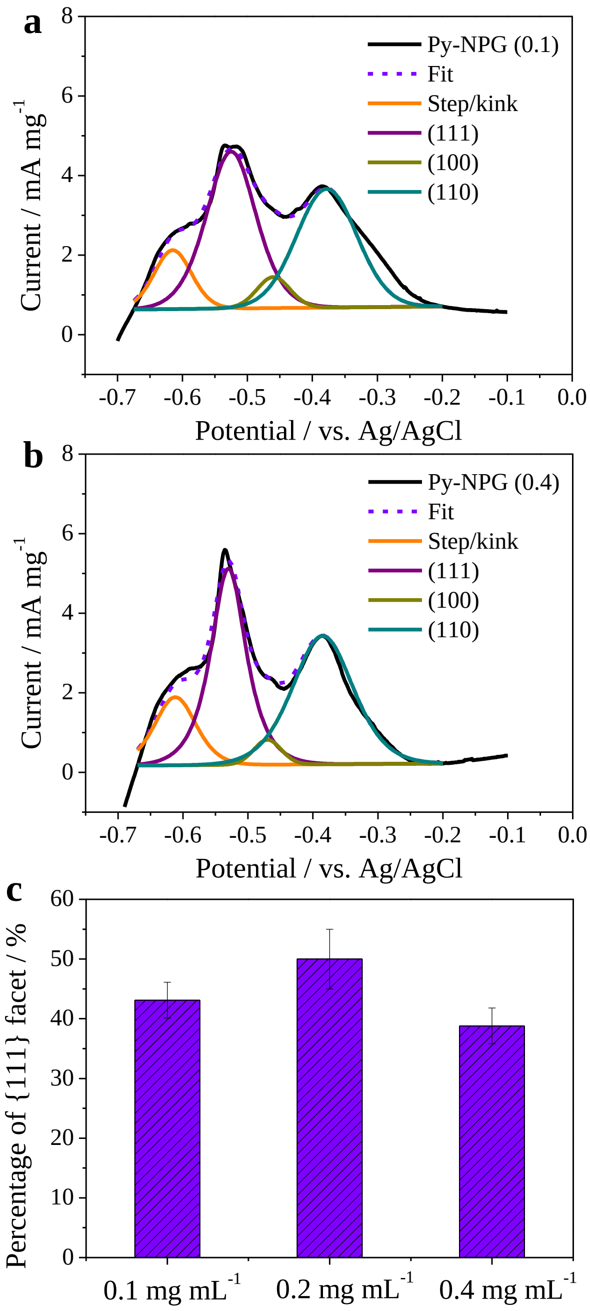

Supplementary Figure 13. Desorption voltammetric profiles of Py-NPG prepared with (a) 0.1 mg mL^{-1} and (b) 0.4 mg mL^{-1} pyrogallol in 0.1 M NaOH + 10^{-3} M $Pb(NO_3)_2$. Scan rate: 50 mV s^{-1}. (c) The percentage of {111} facets versus pyrogallol concentration. Error bars represent standard deviations from three measurements.

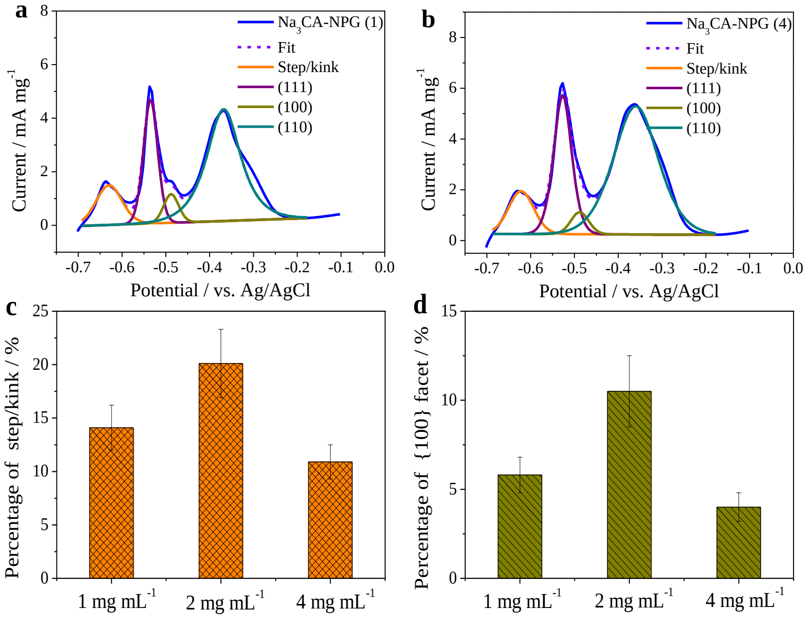

Supplementary Figure 14. Desorption voltammetric profiles of Na_3CA-NPG prepared with (a) 1.0 mg mL^{-1} and (b) 4.0 mg mL^{-1} in 0.1 M NaOH + 10^{-3} M $Pb(NO_3)_2$. Scan rate: 50 mV s^{-1}. The percentages of (c) step/kink sites and (d) {100} facets versus Na_3CA concentrations. Error bars represent standard deviations from three measurements.

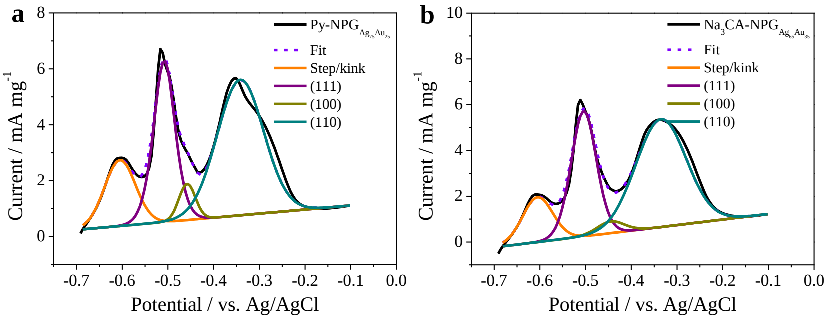

Supplementary Figure 15. Desorption voltammetric profiles of (a) Py-NPG$_{Ag75Au25}$ (using $Ag_{75}Au_{25}$ as precursor) and (b) Na_3CA-NPG$_{Ag65Au35}$ (using $Ag_{65}Au_{35}$ as precursor) in 0.1 M NaOH + 1.0^{-3} M $Pb(NO_3)_2$. Scan rate: 50 mV s^{-1}. The percentage of {111} facets in Py-NPG$_{Ag75Au25}$ is 28.6 %. The percentage of {100} facets in Na_3CA-NPG$_{Ag65Au35}$ is 3.1 %.

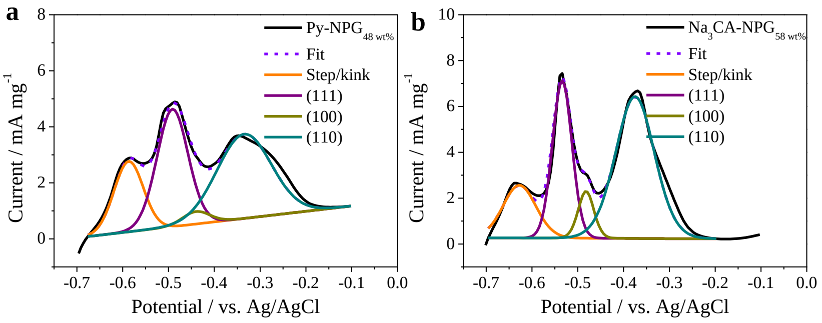

Supplementary Figure 16. Desorption voltammetric profiles of (a) Py-NPG$_{48wt\%}$ (using 48 wt% HNO_3 solution) and (b) Na_3CA-NPG$_{58wt\%}$ (using 58 wt% HNO_3 solution) in 0.1 M NaOH + 10^{-3} M $Pb(NO_3)_2$. Scan rate: 50 mV s^{-1}. The percentage of {111} facets in Py-NPG$_{48wt\%}$ is 34.5 %. The percentage of {100} facets in Na_3CA-NPG$_{58wt\%}$ is 6.6 %.

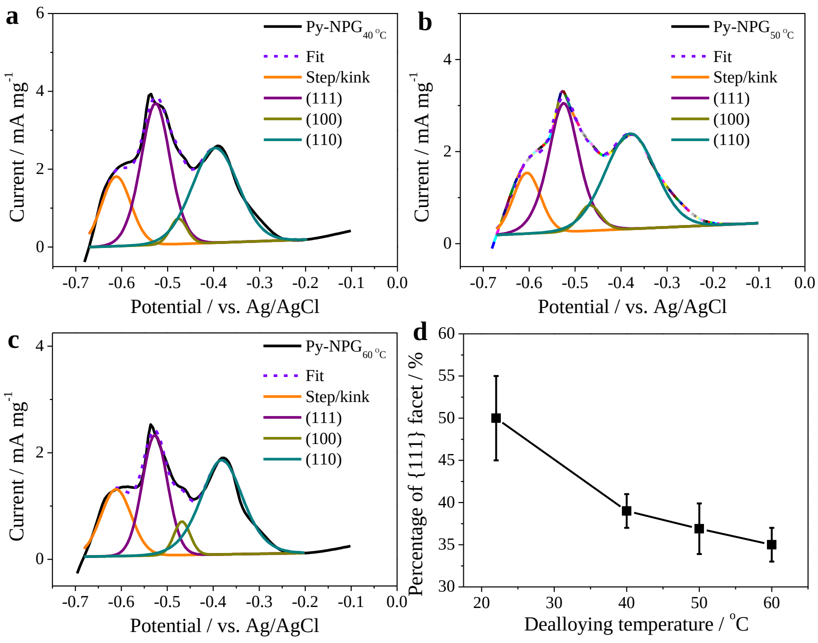

Supplementary Figure 17. Desorption voltammetric profiles of Py-NPG dealloyed at (a) 40 ℃, (b) 50 ℃, and (c) 60 ℃ in 0.1 M NaOH + 10^{-3} M $Pb(NO_3)_2$. Scan rate: 50 mV s^{-1}. (d) The percentage of {111} facets at different dealloying temperature. Error bars represent standard deviations from three measurements.

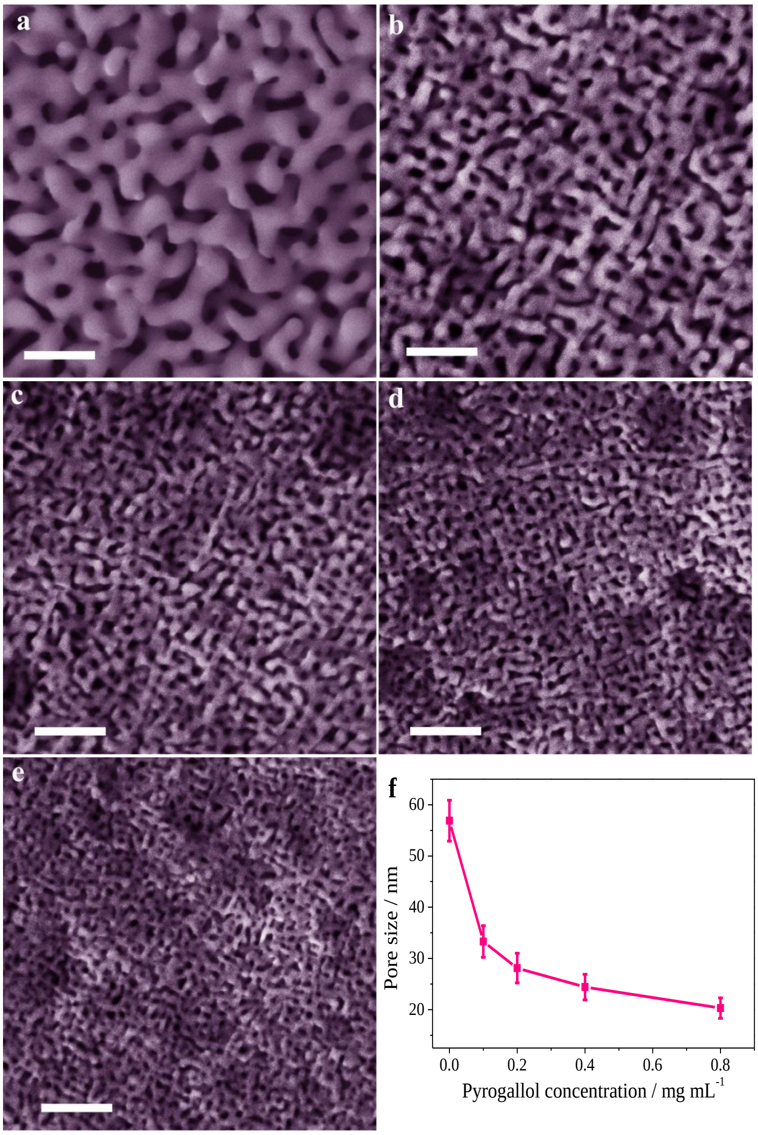

Supplementary Figure 18. SEM images of the NPG samples prepared with different pyrogallol concentrations: (a) 0 mg mL^{-1}, (b) 0.1 mg mL^{-1}, (c) 0.2 mg mL^{-1}, (d) 0.4 mg mL^{-1}, and (e) 0.8 mg mL^{-1}. Scale bars: 200 nm. (f) Pore sizes as function of the pyrogallol concentrations. Error bars represent standard deviations from three measurements.

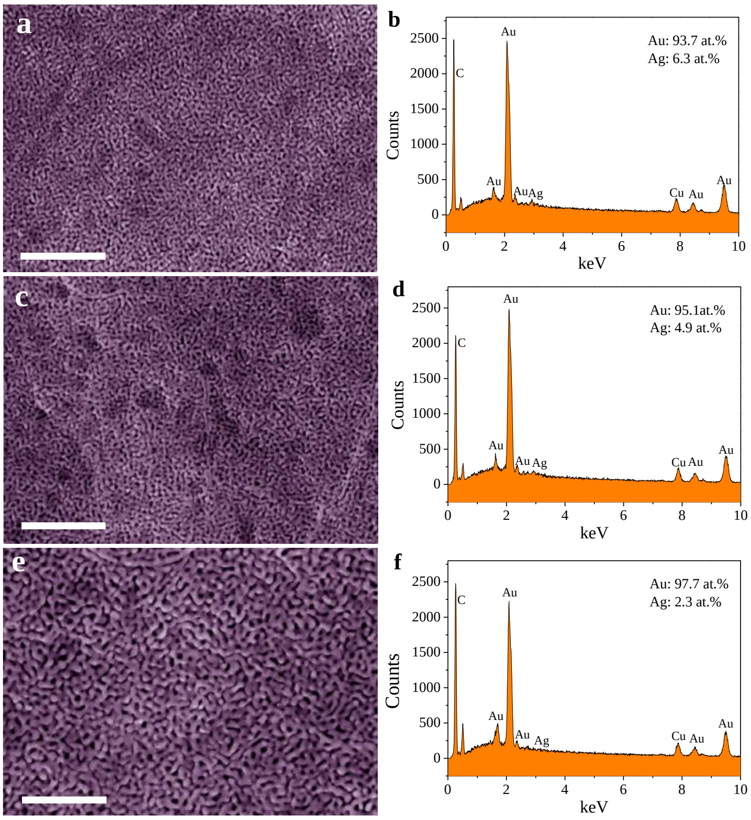

Supplementary Figure 19. SEM images and EDS spectra of (a, b) C-NPG$_9$, (c, d) C-NPG$_{11}$, and (e, f) C-NPG$_{17}$. Scale bars of the SEM images: 500 nm. The Cu peaks in EDS spectra are from the copper sample holder.

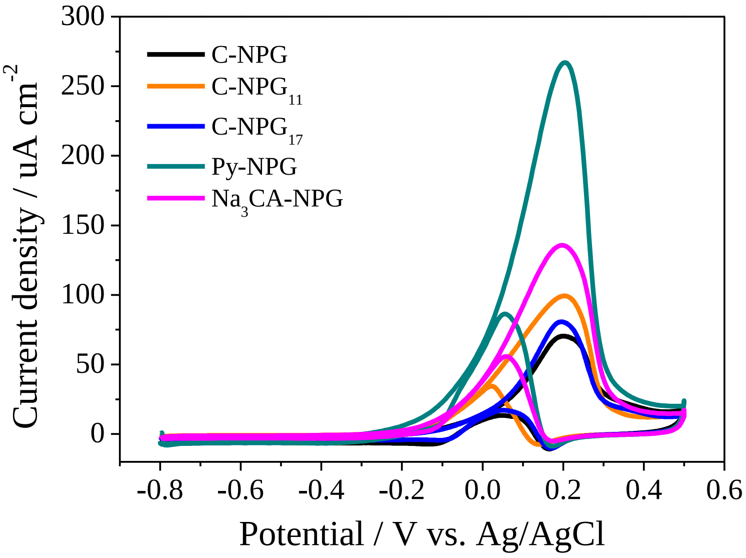

Supplementary Figure 20. CV curves of MOR on C-NPG, C-NPG_{11}, C-NPG_{17}, Py-NPG, and Na_3CA-NPG in 0.5 M KOH/1.0 M methanol solution (scan rate: 10 mV s^{-1}).

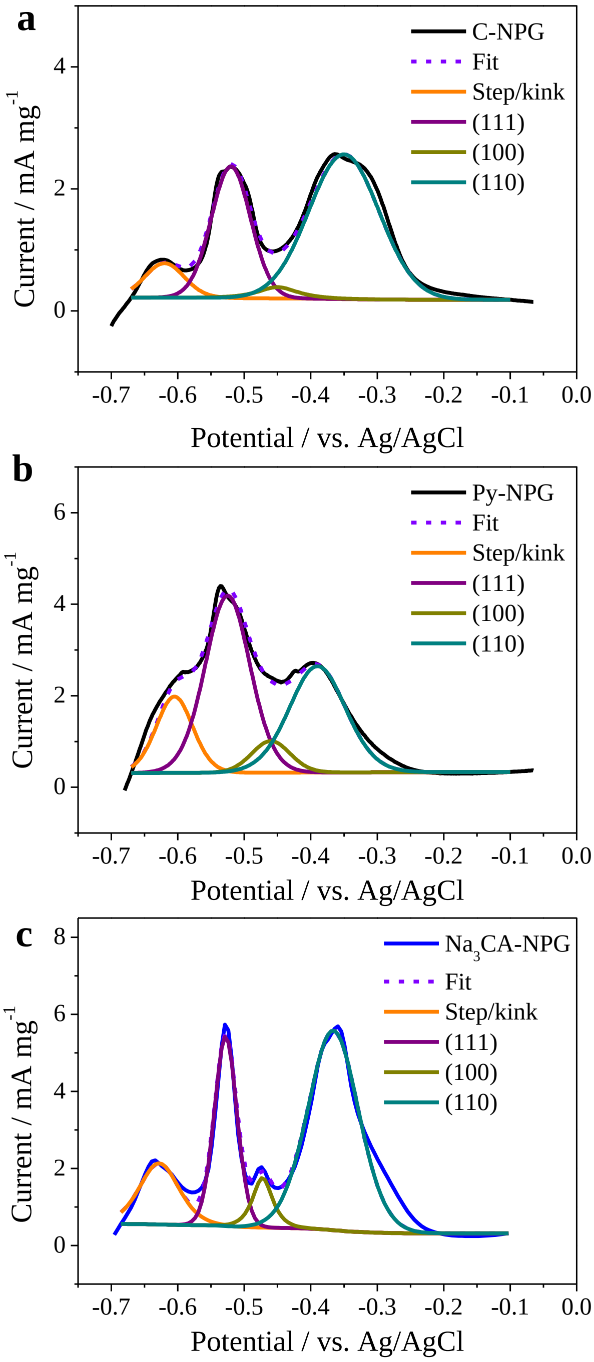

Supplementary Figure 21. Desorption voltammetric profiles of (a) C-NPG, (b) Py-NPG, and (c) Na_3CA-NPG in 0.1 M NaOH + 10^{-3} M $Pb(NO_3)_2$ after CA measurements. Scan rate: 50 mV s^{-1}.

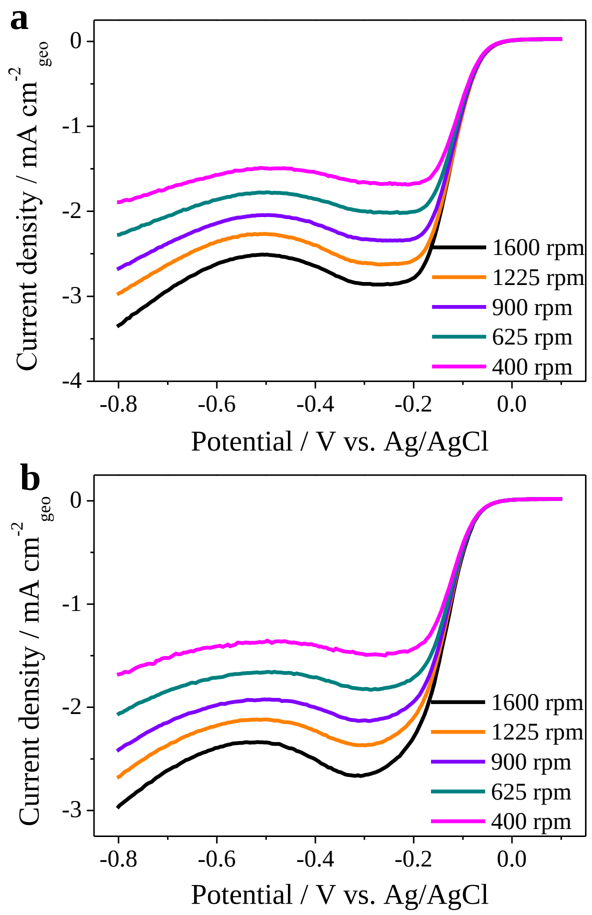

Supplementary Figure 22. ORR polarization curves of (a) Py-NPG and (b) C-NPG at different rotating speeds.

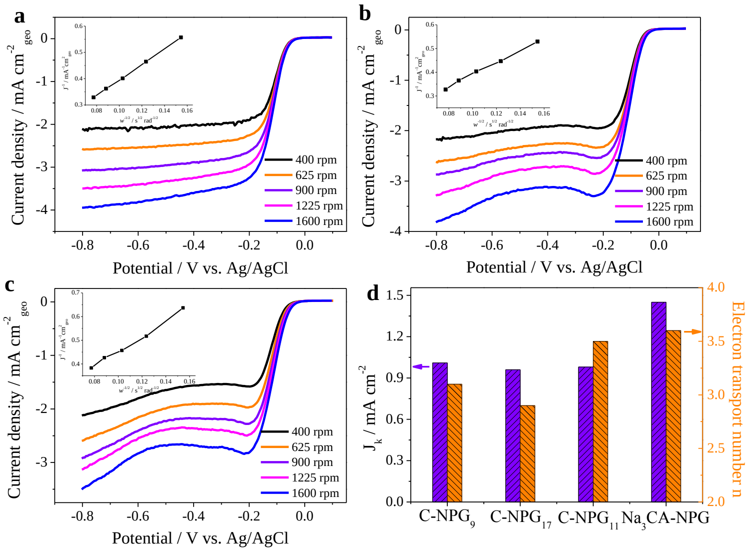

Supplementary Figure 23. ORR polarization curves of (a) C-NPG$_9$, (b) C-NPG$_{11}$ and (c) C-NPG$_{17}$ at different rotating speeds. The insets of (a), (b), and (c) are the corresponding Koutecky-Levich plots at -0.175 V. (d) J$_k$ of different NPG samples as well as the corresponding electron transfer numbers at -0.175 V.

Supplementary Table 1. Chemical compositions of different NPG samples quantified by SEM-EDS and ICP-MS.

Sample	SEM-EDS (Ag/Au)	ICP-MS (Ag/Au)
Py-NPG	2.4/97.6	2.5/97.5
Na_3CA-NPG	3.7/96.3	3.9/96.1
C-NPG	1.6/98.4	1.8/98.2

Supplementary Table 2. Pore sizes and electrochemical active surface areas of different NPG samples.

Samples	Pore size (nm)	Electrochemical active surface area (m^2 g^{-1})
C-NPG	56.9	4.6
Py-NPG	28.1	8.1
Na_3CA-NPG	30.2	7.9
$C-NPG_{11}$	11.2	15.4
$C-NPG_{17}$	17.1	10.3

Supplementary Table 3. The percentages of different facets in C-NPG, Py-NPG, and Na_3CA-NPG before and after CA measurements.

Sample	{111} facets (%) Before/After	{100} facets (%) Before/After	Step/kink (%) Before/After	{110} facets (%) Before/After
C-NPG	29.9/30.2	1.9/2.9	6.3/7.9	61.9/59
Py-NPG	50/45.1	4.7/6.6	14.1/15	31.2/33.3
Na_3CA-NPG	23.4/23.8	10.5/6.0	20.1/13.8	46/56.4
